# New Insights in *Saccharomyces cerevisiae* Response to the Cyanotoxin Microcystin-LR, Revealed by Proteomics and Gene Expression

**DOI:** 10.3390/toxins12100667

**Published:** 2020-10-21

**Authors:** Elisabete Valério, Sara Barreiros, Sara Rodrigues, Maria V. Turkina, Vitor M. Vasconcelos, Alexandre Campos

**Affiliations:** 1 Departamento de Saúde Ambiental, Instituto Nacional de Saúde Doutor Ricardo Jorge, Avenida Padre Cruz, 1649-016 Lisboa, Portugal; sara_estrela_carvalho@hotmail.com (S.B.); sara.cris.rodrigues@gmail.com (S.R.); 2 Department of Biomedical and Clinical Sciences, Faculty of Medicine and Clinical Sciences, Linköping University, 581 83 Linköping, Sweden; maria.turkina@liu.se; 3 Centro Interdisciplinar de Investigação Marinha e Ambiental (CIIMAR/CIMAR), University of Porto, Terminal de Cruzeiros do Porto de Leixões, Av. General Norton de Matos, s/n, 4450-208 Matosinhos, Portugal; vmvascon@fc.up.pt (V.M.V.); acampos@ciimar.up.pt (A.C.); 4 Biology Department, Faculty of Sciences, University of Porto, Rua do Campo Alegre, s/n, 4169-007 Porto, Portugal

**Keywords:** Microcystin-LR, *Saccharomyces cerevisiae*, MTT assays, shotgun proteomics, BER genes, Real-Time RT-qPCR

## Abstract

Microcystins (MCs) are hepatotoxins produced by some cyanobacteria. They are cyclic peptides that inhibit the serine/threonine protein phosphatases (PPs) PP1 and PP2A, especially PP2A. The inhibition of PP2A triggers a series of molecular events, which are responsible for most MC cytotoxic and genotoxic effects on animal cells. It is also known that MCs induce oxidative stress in cells due to the production of reactive oxygen species (ROS). However, a complete characterization of the toxic effects of MCs is still not accomplished. This study aimed to clarify additional molecular mechanisms involved in MC-LR toxicity, using *Saccharomyces cerevisiae* as eukaryotic model organism. First, a shotgun proteomic analysis of *S. cerevisiae* VL3 cells response to 1 nM, 10 nM, 100 nM, and 1 μM MC-LR was undertaken and compared to the control (cells not exposed to MC-LR). This analysis revealed a high number of proteins differentially expressed related with gene translation and DNA replication stress; oxidative stress; cell cycle regulation and carbohydrate metabolism. Inference of genotoxic effects of *S. cerevisiae* VL3 cells exposed to different concentrations of MC-LR were evaluated by analyzing the expression of genes *Apn1*, *Apn2*, *Rad27*, *Ntg1*, and *Ntg2* (from the Base Excision Repair (BER) DNA repair system) using the Real-Time RT-qPCR technique. These genes displayed alterations after exposure to MC-LR, particularly the *Apn1*/*Apn2*/*Rad27*, pointing out effects of MC-LR in the Base Excision Repair system (BER). Overall, this study supports the role of oxidative stress and DNA replication stress as important molecular mechanisms of MC-LR toxicity. Moreover, this study showed that even at low-concentration, MC-LR can induce significant changes in the yeast proteome and in gene expression.

## 1. Introduction

Microcystins (MCs) are potent hepatotoxins produced by some cyanobacteria, especially by the species *Microcystis aeruginosa*. Many different chemical variants have been reported so far [1], being MC-LR, which has the amino acids leucine (L) and arginine (R) in the positions 2 and 4 of its structure, the variant most commonly found and also one of the most prevalent cyanotoxin in freshwaters and consequently the most studied one. The increased prevalence of this group of toxins in the environment is likely associated to its synthesis that can be carried out by different genera of cyanobacteria [2], some of which have a cosmopolitan distribution. Moreover, MCs are also relatively stable and resistant to degradation in water [2].

The molecular mechanisms behind MC-LR toxicity, especially during chronic exposure, are complex and not completely clarified [3,4]. The knowledge gathered on this topic, so far, was object of recent reviews [3,4,5,6]. The main aspect is that MC-LR has high affinity for serine/threonine protein phosphatases 1 (PP1) and 2A (PP2A) and binds to the catalytic subunit of these enzymes, acting as their inhibitors, especially of PP2A [3]. Another toxic effect of MC-LR is the induction of reactive oxygen species (ROS) and glutathione (GSH) depletion [7,8,9,10]. Almost all organisms have antioxidant defense systems but a continuous exposure to MC-LR may disrupt it and lead to the depletion of GSH [7]. Regarding the mechanism of ROS production, Ding and Nam Ong (2003) proposed that MC-LR increased the oxidative stress by two primary pathways: first by leading to a depletion of GSH and secondly by disrupting the electron transport in the mitochondria. Both mechanisms can result in oxidative damage and cell death by apoptosis [11]. The oxidative stress, which can cause cell death by apoptosis or necrosis, is related with the mitochondria metabolism [3,4].

Ji and co-workers (2011) showed that MC-LR stimulated nitric oxide (NO) production in a dose-dependent manner in rat insulinoma (INS-1) cells [12]. Therefore, nitric oxide (NO) may also be considered an important factor of MC-LR toxicity. The results of Wang et al. (2015) in human-hamster hybrid cells exposed to MC-LR, showed that the genotoxicity associated with chronic MC-LR exposure in mammalian cells was mediated by NO [13]. Furthermore, a recent study showed that MC-LR induced apoptosis via S-nitrosylation of GAPDH in colorectal cancer cells through a molecular mechanism of NO/GAPDH/Siah1 cascade [14] thus supporting the role of NO in MC-LR toxicity.

Our previous studies have described toxic effects of MC-LR in *S. cerevisiae*. These studies showed, for instance, that MC-LR induced an increase of H_2_O_2_ in *S. cerevisiae* as the main ROS [15]. Moreover, signs of apoptosis and some cell death by necrosis were also detected after exposing the yeast cells to MC-LR [15], consistent with the responses observed in mammal cells exposed to MC-LR [16]. Furthermore, a set of 14 proteins affected by MC-LR were revealed by proteomics. The proteins identified were involved in metabolism, genotoxicity, cytotoxicity and stress response [17]. In fact, proteomics, along with other OMICs disciplines (Genomics and Metabolomics), has been an extremely useful tool to understand the mode of action of toxic compounds like MC-LR. Among the proteomics studies developed on MC-LR toxicity, we highlight for instance the chronic toxicity of MC-LR (1, 10 and 100 µg/L) in mice testis, with Isobaric Tag for Relative and Absolute Quantitation (iTRAQ) methodology [18]. The molecular processes affected by this toxin were related with biological adhesion, cellular process, response to stimulus, or rhythmic process. It was also denoted a possible dysfunction of blood-testis barrier (BTB) due to alterations in proteins from tight junctions and gap junctions [18]. Other effects revealed by proteomics were related to ribosome activity and gene translation, energy metabolism and oxidative phosphorylation [19]. Interestingly, proteomics enabled to reveal changes in the proteome at the sub-toxic level, i.e., when MC-LR concentrations are below the No Observed Adverse Effect (NOAEL) [20].

*S. cerevisiae* has two major DNA repair systems: Nucleotide Excision Repair (NER) and Base Excision Repair (BER). The NER pathway can remove structures that interfere with base pairing and transcription [21]. The BER pathway removes most of the nucleotides damaged by oxidative DNA lesions to deamination [22]. We can hypothesize that ”since MC-LR is able to induce an increase of the ROS levels inside the cell, it is expected that these may then lead to oxidative DNA damage that will be subsequently repaired by the DNA BER system”. We would like to address this hypothesis with this study.

Formerly, when we performed preliminary analysis of the relative expression of yeast’s genes PP2A and also of *Ntg1* and *Ntg2* (from BER DNA repair system), we observed more alterations of these genes at 1 nM MC-LR, opposing to the ones observed with higher MC-LR concentrations (100 nM and 1 uM) [17]. This dependence on the strength of the stimulus, i.e., the dose and/or the exposure time, was also previously observed by Humpage and Falconer (1999) [23]. The authors reported that cytokinesis was stimulated and the rate of apoptosis reduced in the presence of picomolar concentrations of MC-LR. At higher nanomolar concentrations, cytokinesis was inhibited and cell death was induced [23]. In Vero-E6 cells, MC-LR also showed a dual effect in the stimulation of cell proliferation: induction of autophagy/apoptosis or ultimately necrosis highly dependent on the dose and/or the exposure time [16,24,25,26].

In this study, we aimed to gain more insights regarding the molecular mechanisms involved in MC-LR toxicity, using *S. cerevisiae* as eukaryotic model organism. Cytotoxicity was evaluated using MTT (methyl-thiazolyl-tetrazolium) assays, one of the most commonly used tests to evaluate cells viability. To identify the proteins altered by exposure to MC-LR, a shotgun proteomic analysis of *S. cerevisiae* VL3 cells response to 1 nM, 10 nM, 100 nM, and 1 µM MC-LR was undertaken and compared to the control (cells grow in Yeast Extract–Peptone–Dextrose (YPD) medium, without MC-LR). The genotoxic effects of *S. cerevisiae* VL3 cells were inferred by analyzing the gene expression profiles of genes *Apn1*, *Apn2*, *Rad27*, *Ntg1* and *Ntg2* (from the BER DNA repair system) using the Quantitative Real-Time RT-PCR (Real-Time RT-qPCR) technique.

## 2. Results

### 2.1. Saccharomyces cerevisiae Cells Viability When Exposed to Different Concentrations of MC-LR

The MTT assays were conducted with *S. cerevisiae* commercial strain VL3. Two positive controls were used (Hydrogen peroxide, H_2_O_2_ and Sodium Dodecyl-Sulphate, SDS) which showed significant differences (*p* ≤ 0.05) from the control (cells grown in YPD). We obtained a 59.4% decrease in relative cell viability in cells treated with 50 mM H_2_O_2_ and a decrease of 89.2% when 5% SDS was added to the medium (Table 1). These results confirm that the MTT method is suitable to report cell viability in *S. cerevisiae* cells. However, when the MTT viability assays were used to asses cytotoxic effects of the different MC-LR concentrations (exposed during 4 h), they did not reveal significant cytotoxic effects in *S. cerevisiae* (*p* > 0.05) (Table 1).

### 2.2. Proteomic Analysis

#### 2.2.1. Protein Expression

**Figure 1:**
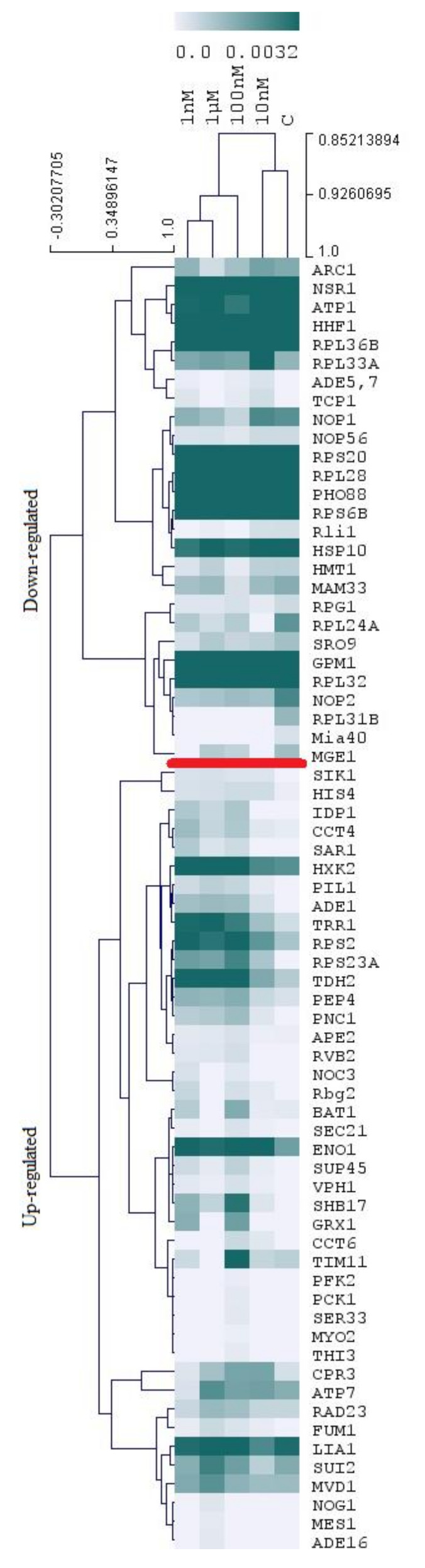
Color map and cluster analysis of differentially expressed proteins (DEPs) from yeast subjected to different concentrations of MC-LR. Column labels (1 nM, 10 nM, 100 nM, and 1µM) identify the different experimental groups and control (C). Row labels are abbreviations of protein names. Relative expression of proteins (average values varying between 0.0 and 0.0032) are disclosed in a color gradient. Up- and down-regulated protein groups are separated by a red line. Detailed information concerning protein names, expression levels, and statistical analysis (ANOVA, *p* < 0.05) are reported in Supplementary Table S2.

Proteomics analysis (shotgun approach) led to the identification of 738 proteins from a total of 15 samples analyzed (five experimental groups). The names and expression values of these proteins are reported in Supplementary Table S1. Statistics (ANOVA test) revealed changes in protein expression with exposure to MC-LR toxin. Overall, 69 proteins were significantly up- or down-regulated after exposure to four concentrations of this cyanotoxin. The expression of these proteins is reported in Figure 1, using a color gradient to represent expression levels (heatmap). All information regarding the relative expression of these proteins and statistical analysis can be found in the Supplementary Table S2. We verified that more proteins were up-regulated (43 proteins) and less down-regulated (26 proteins). Cluster analysis of the various experimental groups (MC-LR concentrations, to which the cells where exposed to) (Figure 1, top cluster analysis) clearly separates the control group from the group exposed to the highest concentration of the toxin (1 µM). This indicates that there are more differences in the protein expression profiles between these two sample groups, than between control and 10 nM group or between 1 nM, 10 nM, and 100 nM groups. This result indicates that 1 nM, 10 nM, and 100 nM groups display less differences in protein expression (Figure 1).

#### 2.2.2. Protein Functional Analysis

Functional analysis of all differentially expressed proteins between all sample groups was performed using the Search Tool for Retrieval of Interacting Genes (STRING) [27]. This program performs database analysis of protein interactions. From the network of protein interactions predicted by the program, it is possible to infer the molecular processes and pathways in which proteins are involved. Molecular processes can be revealed as subsets of proteins (protein clusters) displaying dense interaction networks in comparison to the background proteome network.

**Figure 2:**
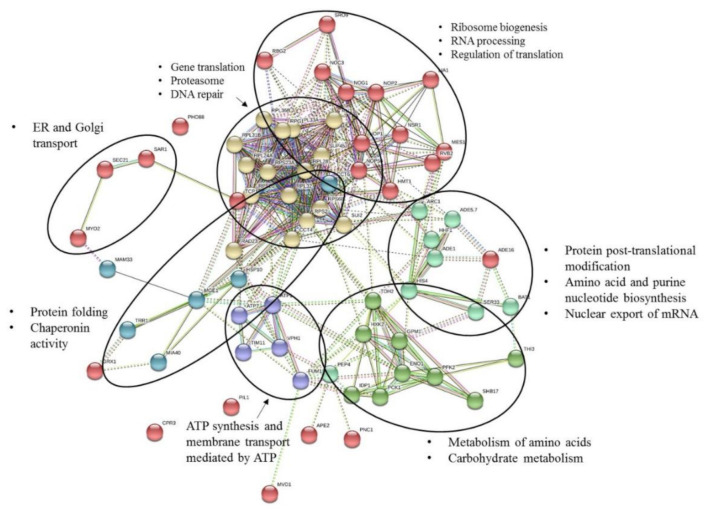
Predicted protein functional associations, from STRING database analysis. The evidences supporting these associations are represented by edges of different colors (detailed information regarding the meaning of the color codes of edges, is found in the program page https://stringdb.org/). Within the interacting network, functionally related proteins are organized in clusters and have same color. The elements of a cluster are delimited by a circle. Moreover, the molecular processes that characterize each cluster are also reported in the figure. The STRING analysis was run with medium confidence (interaction score 0.4).

This analysis revealed a complex protein interaction network, suggesting multiple functional relationships between the proteins affected by MC-LR toxin (Figure 2).

Several functional clusters are identified in this network, being the largest cluster (the one with more elements) represented by several ribosomal proteins (e.g., RPS23A, RPS20, RPS2, RPS6B, RPL32, RPL33A, RPL24A) and several proteins with functions in the regulation of gene translation (e.g., SUP45, RLI1, SUI2, RPG1), proteasomal degradation of misfolded proteins and DNA repair (RAD23) (Figure 2, proteins with yellow color). This cluster establishes interactions with proteins from another cluster (red color) that integrate several proteins with functions related with ribosome biogenesis (NOP56, NOC3), pre-rRNA processing (NOP1, NOP2, NSR1), regulation of translation (LIA1, SRO9), mRNA processing and export (HMT1) and polypeptide synthesis (MES1).

Another protein cluster (proteins with aquamarine color) includes proteins from processes related with protein synthesis and post-translational modification (ARC1, PEP4) amino acid and purine nucleotide biosynthesis (SER33, ADE1, ADE5,7), nuclear export of spliced and unspliced mRNA (BAT1) and histones (HHF1, HIS4).

The fourth cluster found by this analysis (proteins in green) include proteins with functions in the metabolism of amino acids (THI3) and carbohydrate metabolism, i.e., from TCA cycle and glycolysis (PCK1, TDH2, PFK2, IDP1, GPM1, HXK2, ENO1, SHB17).

The analysis revealed also two other clusters (proteins in blue and purple) constituted by proteins involved in protein folding and chaperoning activity (HSP10, MGE1, CCT6, MIA40), cell redox homeostasis and oxidative stress (TRR1), proteins involved in DNA repair in response to DNA damage (FUM) and ATP synthesis and membrane transport mediated by ATP (ATP7, ATP1, VPH1, TIM11).

Other proteins showed to be poorly related with these clusters and established few interactions with proteins from the clusters identified. These include PNC1, APE2, MVD1, CPR3, GRX1, that are proteins related with cofactor biosynthesis, proteolysis, isoprenoid biosynthesis, protein folding, cell redox and cellular response to oxidative stress, respectively.

The link between MYO2, SAR1 and SEC21, revealed in the network, reflect the participation of these proteins in common pathways, such as in endoplasmic reticulum (ER) and golgi transport and cell cycle-regulated transport of organelles and proteins.

In Table 2, we report the functions of key protein markers of the processes affected by MC-LR, as well as the toxin concentrations that induced their expression alteration. The table shows that most of these proteins were affected by more than one MC-LR concentration.

### 2.3. Relative Gene Expression Alterations of BER Genes in Saccharomyces cerevisiae Exposed to Different MC-LR Concentrations

**Figure 3:**
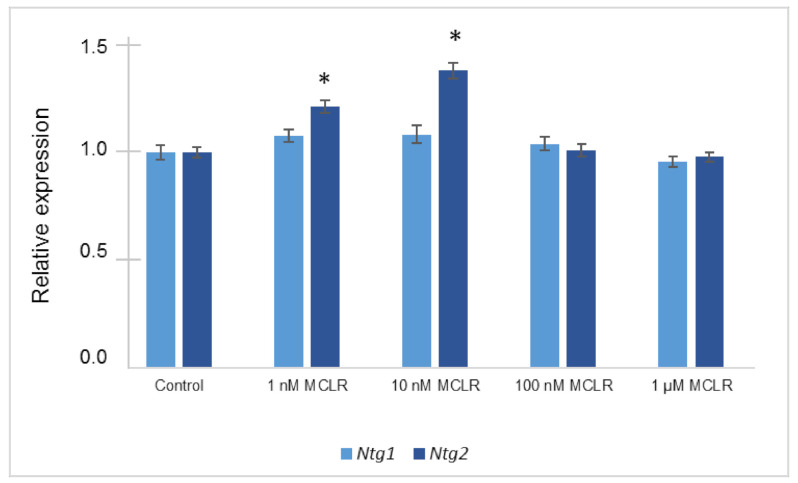
Relative expression levels of *Ntg1* and *Ntg2* genes of *S. cerevisiae* VL3 cells exposed to 1 nM, 10 nM, 100 nM, and 1 µM of MC-LR compared to the control (cells grown in YPD, without MC-LR). Results are expressed as the mean ± standard deviation of five independent biological experiments. Asterisks (*) represent statistically significant differences between the cells exposed to MC-LR and the control cells (*p* < 0.05; *t*-Test).

The expression results of *Ntg1* and *Ntg2* genes of the BER system are displayed in Figure 3. In the case of *Ntg1* the presence of MC-LR did not revealed significant differences from the control, although MC-LR at 1 nM and 10 nM led to a slight, but not significant, over-expression of this gene. The *Ntg2* gene displayed similar alterations but showed significant statistical differences from the control in the presence of 1 and 10 nM MC-LR (*p* < 0.05).

**Figure 4:**
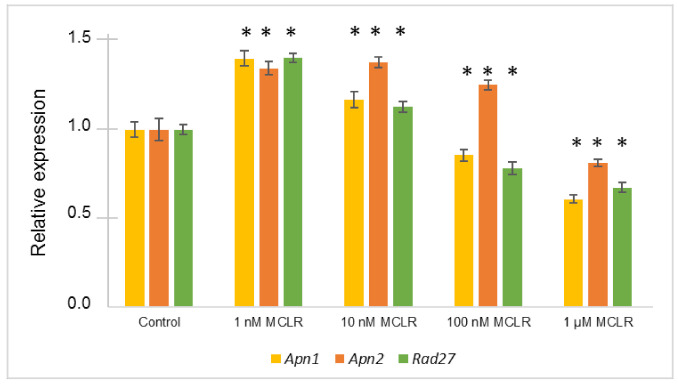
Relative expression levels of *Apn1*, *Apn2,* and *Rad27* genes of *S. cerevisiae* VL3 cells exposed to 1 nM, 10 nM, 100 nM, and 1 µM of MC-LR compared to the control (cells grown in YPD, without MC-LR). Results are expressed as the mean ± standard deviation of five independent biological experiments. Asterisks (*) represent statistically significant differences between the cells exposed to MC-LR and the control cells (*p* < 0.05; *t*-Test).

*Apn1*, *Apn2,* and *Rad27* genes of the BER system were significantly altered in the presence of MC-LR, however displayed different expression profiles (Figure 4). There was an up-regulation of *Apn1* and *Rad27* genes in response to the lower doses of MC-LR tested (1 nM and 10 nM). On the contrary, for the higher doses (100 nM and 1 µM), there is a tendency for the repression of these genes. The *Apn2* gene showed an induction in the presence of 1 nM, 10 nM, and 100 nM of MC-LR, only being repressed with the highest dose tested (1 µM).

## 3. Discussion

The MTT assay is a method to determine cell viability through the reduction of 3-(4,5-dimethyl-2-thizaolyl)-2,5-diphenyl-2H-tetrazolium bromide into a colored product, formazan, by mitochondria dehydrogenases to which the cell membrane is impermeable [28]. The MTT viability assays were used in this study to assess cytotoxic effects MC-LR on yeast cells, revealing no significant cytotoxic effects in *S. cerevisiae* VL3 (*p* > 0.05).

To verify if the absence of cytotoxic effects was related to some insensitivity problem of the VL3 strain, we tested a second strain: *S. cerevisiae* VR5 (Fermicru). The results obtained with this second strain were similar (data not shown), thus confirming that the MTT assay does not reveal any cytotoxic effect when the *S. cerevisiae* cells are exposed to MC-LR. There are not many studies that use the MTT method with *S. cerevisiae*, nevertheless, the MTT assays are able to display decreases of *S. cerevisiae* viability as verified with the positive controls used (H_2_O_2_ and SDS).

Previous studies, using MTT assays, revealed that there is a negative correlation between cell viability and MC-LR concentration, where there is a significant dose/time dependent cytotoxic effect of MC-LR on cell viability [28,29]. However, these studies were applied to mammal cell lines and used considerably higher concentrations of toxins than the ones tested in this study [28,29].

The proteomic analyses performed revealed a complex pattern of responses in yeast cells exposed to MC-LR, involving several molecular processes. With regard to our previous proteomics work [17], we denote the increased analytical capacity of the shotgun methodology over Two-dimensional gel Electrophoresis (2DE) based proteomics to characterize changes in yeast proteome. In our previous work, we were able to detect expression differences of only 14 proteins and functions assigned to only six of them. With the current approach, we were able to characterize in more detail the changes in the proteome of yeast cells exposed to MC-LR.

From the results obtained, it may be inferred that the molecular process most affected by the presence of MC-LR was gene translation. Indeed, the expression of many ribosomal proteins and proteins related with RNA processing, ribosome biogenesis and regulation of translation were affected (Figure 2). While some of these proteins were down-regulated (RPS6B, RPS20, RPL32, RPL24A, HMT1, NSR1, NOP2), some others were up-regulated (RPS2, RPS23A, RPL36B), making difficult to assess the overall effects of the toxin, and to infer if gene translation was inhibited or increased during yeast exposure to the toxin. However, the decrease in the expression of the translation initiation factors RLI1 and RPG1 (Table 2 and Supplementary Table S2), may anticipate a negative effect of this toxin in gene translation. RLI1 in particular, has shown to be a primary cellular target of ROS, and indeed impairment of the functions of this protein were previously related with increase of ROS toxicity [30]. Both proteins are essential to gene translation. RLI1 is involved in ribosome biogenesis and maturation, translation initiation and ribosome recycling [30] and RPG1 has a key role in the recruitment of pre-initiation complex (PIC) to mRNA [31]. Moreover, alterations in histones and RAD23 suggest the occurrence of alterations in chromatin and DNA caused by MC-LR. RAD23 was up-regulated at high MC-LR concentrations (100 nM and 1 µM) (Table 2). This protein is involved in proteasome-mediated degradation of misfolded proteins in the endoplasmic reticulum, but also plays a primary function in DNA repair, by supposedly mediating RAD4 stabilization and recognition of DNA damaged sites, in NER mechanism [32]. The repression of this protein has been related with increased sensitivity of cells to DNA-damaging agents [32]. Previously, we detected the increase of the intracellular ROS levels when *S. cerevisiae* was exposed, in similar conditions, to MC-LR [15]. Increase of ROS was verified even for the lowest MC-LR concentration (1 nM) [15]. Our previous results thus support our current hypothesis that MC-LR caused an increase in intracellular ROS. This ROS increase would explain, for instance, the down-regulation of RLI1 protein. On the same note, the increase in ROS would also explain the increase in the expression of thioredoxin reductase 1 (TRR1). This protein was up-regulated in yeast cells exposed to 100 nM and 1 µM MC-LR (Table 2). Regarding protein functions, TRR1 play a key role in the removal of superoxide radicals and thereby in maintenance of cell redox homeostasis [33].

Furthermore, ROS generation is known to be intrinsically related with mitochondria metabolism [34]. Two mitochondrial proteins, HSP10 and MGE1, were down-regulated with the exposure to the toxin (Table 2), which may indicate that mitochondria functions were affected. HSP10 is essential in protein biogenesis in mitochondria. MGE1 has also chaperonin activity and plays an important role in protein import into mitochondrial matrix. Our results are still insufficient to ascertain the involvement of mitochondria in the response of yeast to MC-LR. Notwithstanding, the role of this organelle in yeast toxicity should not be disregarded, and indeed several effects observed previously, such as the increase of ROS and the nuclear alterations like chromatin condensation and fragmentation, suggestive of apoptotic mechanisms [15], can be explained in light of the mitochondrial functions.

The effects of MC-LR in PP1 and PP2A, should be also examined and discussed, since these proteins exert a profound influence in the proteome and in the whole metabolism of cells and are specific targets of this toxin [35]. Our proteomic results do not evidence any direct alterations in PP1 and PP2A. However, our results revealed alterations in several proteins that are known to interact with PP1 and PP2A, thus giving indirect evidences that PPs can be involved in the alterations observed in yeast proteome. For instance, CCT4 was up-regulated by MC-LR (Table 2). This protein is a subunit of T-complex protein 1 (TCP1). TCP1 play chaperonin functions, i.e., participates in the folding of proteins in a process dependent of ATP. It was also demonstrated that this complex interacts with PP2A [35], being this phosphatase an important regulator of TCP1 functions. According to the recent study describing the human protein phosphatase interactions and dynamics, several other proteins identified in this work can be considered putative PP1 and PP2A interacting targets and thus be regulated by these phosphatases. Among these are the ribosomal proteins (RPL and RPS), RLI1, PGM1 and also the heat shock proteins (HSPs).

Finally, exposure to MC-LR seems also to affect carbohydrate metabolism. Several glycolytic and TCA cycle proteins were altered (PCK1, TDH2, PFK2, IDP1, GPM1, HXK2, ENO1, and SHB17). In particular, the up-regulation of TDH2, IDP1, ENO1 and SHB17 (Figure 1, Supplementary Table S2), suggest that these processes were activated in yeast cells. The increase of TCA and glycolysis may be required to increase ATP pool in yeast cells, and energy to cope with the toxic effects of MC-LR.

In light of the results observed from the MTT test and the proteome, that in general do not evidence acute adverse effects, we hypothesize that the MC-LR concentrations tested are sub-toxic or sub-lethal to yeast cells, as cells did not shown any decrease in viability in the range of concentrations tested, as verified when using the MTT test.

Regarding gene expression obtained by Real-Time RT-qPCR, we denote that *Ntg1* and *Ntg2* expression results differ in some extent from the results observed in our previous study [17]. No variations were observed in *Ntg1* contrasting with the previous over-expression of this gene at 1 nM and the decrease in expression at 100 nM and 1 µM. However, a consistent increase in *Ntg2* expression was verified in both studies for MC-LR concentrations between 1 nM and 100 nM. The differences in the expression of *Ntg1* between studies are difficult to explain, and apparently are not related with the experimental conditions since these were similar in both studies.

The gene expression levels analyzed by Real-Time RT-qPCR showed that the lower MC-LR concentrations (1 nM and 10 nM) are the ones that resulted in an up-regulation of all the BER system genes. This is in accordance with the explanation that induction of genes expression may contribute to overcome the effects of the toxin when its concentration is low. However, at high toxin concentrations some genes (*Apn1* and *Rad27*) were repressed, which may suggest that the DNA had extended deleterious effects that could not be fully overcome by this repair mechanism.

A previous study compares yeast life span with BER activity [36]. It shows that losses of enzymes Ntg1p and Ntg2p increase both spontaneous and hydrogen peroxide induced mutation frequencies, though it generally does not cause cell death. A single deletion of these enzymes showed little or no effect in life span, however, combined deletions resulted in a decreased cell survival [36]. Moreover, in this study, we did not observe cell death (using MTT test) despite the alterations in *Ntg1* and *Ntg2* genes.

Apn1p is considered the most important apurinic/apyrimidinic (AP) endonuclease. Without this enzyme the spontaneous mutation rate would increase, becoming more sensitive to hydrogen peroxide. The Apn2p seems to confer resistance to ROS induced damages in an alternative pathway to the Apn1p. The combined loss of both endonucleases exhibited extremely poor cell survival [36]. Our results suggest that MC-LR is able to affect the BER DNA repair system mechanism, particularly the *Apn1*/*Apn2*/*Rad27* genes. These results are consistent with the hypothesis that the induction of ROS is an important molecular mechanism of MC-LR genotoxicity, and also gives more insights that H_2_O_2_ is a relevant ROS induced.

It should also be noted that MC-LR concentration of 1 nM, proposed by World Health Organization (WHO) as guideline value for drinking water, induced a significant change in a high number of proteins and also in the gene expression levels of the BER system (real-time results). This agrees with the finding of He and co-workers [20] where most significant alterations in the proteome were observed in low-concentrations of MC-LR.

Unfortunately, we were not able to corroborate the gene expression results at the protein level. We verify that the corresponding gene products were not present in the sub-set of the yeast proteome identified by the shotgun method (Supplementary Table S1). The reason behind the absence of these proteins from our proteomic dataset can be due to their expression in the cell that may be below the detection limits of the proteomics methodology or were underrepresented (e.g., low extraction efficiency of these proteins). RAD23 was the only protein identified whose functions can be linked to DNA repair and to the NER system. The molecular functions of this protein, however, is different from RAD27, which may explain the differences in expression verified at the gene (RAD27) and protein (RAD23) levels. However, taken together, the results attained in *Apn1*, *Apn2*, *Rad27, Ntg2,* and RAD23 strongly suggest that the toxin affects DNA integrity and leads to responses involving DNA repair systems.

## 4. Conclusions

In this work, we present new data concerning *Saccharomyces cerevisiae* VL3 response to the cyanotoxin MC-LR, revealed by proteomics and gene expression. This simple eukaryotic organism has been widely used as a eukaryotic cell model and has been particularly helpful to explore the molecular mechanisms and affected pathways of several compounds in toxicology.

The proteomics study revealed significant changes in the yeast proteome induced by this cyanotoxin. The main proteins alterations here observed were associated to gene translation, DNA replication stress; oxidative stress; cell cycle regulation and carbohydrate metabolism. Alterations in specific proteins, such as RLI1, TRR1, and RAD23, for instance, suggest an increase of ROS in yeast cells and effects them at the DNA level.

The assessment of genotoxic effects exploited using Real-Time RT-qPCR assays showed that MC-LR affects the genes of BER DNA repair system mechanism, supporting the existence of genotoxic effects induced by MC-LR, caused by intracellular ROS levels increase.

The results of this study further support that current guidelines for MCs in drinking water may need a revision since chronic exposure to low doses of MC-LR and associated risks to human health are currently still not fully elucidated.

## 5. Materials and Methods

### 5.1. Saccharomyces cerevisiae Cultures

*S. cerevisiae* VL3 (Zymaflore, Bordeaux, France) pre-culture was left growing overnight in YPD medium (1% yeast extract, 2% peptone, 2% glucose) at 20 °C, 300 rpm. Then, 20 mL culture was inoculated in five 150 mL flasks with a cell density adjusted to an initial optical density DO_600nm_ = 0.05, corresponding to approximately 5.5 × 10^5^ cells/mL [37,38]. Each flask was treated with different pure MC-LR concentrations: without toxin (control), 1 nM, 10 nM, 100 nM, and 1 µM. The cultures were left growing for 4 hours at a climatic chamber at 20 ± 2 °C and 300 rpm (shaker IKA Labortechnik, IKA, Staufen, Germany). This exposure time was selected by our results previously obtained [15], where we observed that after 4h the cells started to recover, reaching approximately the same OD as the control.

Cells were harvested in 15 mL plastic tubes (Sarstedt) by centrifugation at 834× *g* on a high capacity centrifuge (Sorvall RT 6000D, Dupont, Wilmington, DE, USA) for 3 min and washed with 1 mL of RNase free sterile water (Gibco, Carlsbad, CA, USA). Cells were transferred into 1.5 mL tubes and centrifuged (Eppendorf 5415C Centrifuge, Eppendorf, Hamburg, Germany) for 3 min at 8000× *g*. The supernatant was discarded and the pellet was immediately frozen in liquid nitrogen and kept at −80 °C until use.

### 5.2. Analysis of Saccharomyces cerevisiae Viability When Exposed to Different Concentrations of MC-LR

To evaluate cells viability when exposed to different chemical compounds and MC-LR, MTT (methyl-thiazolyl-tetrazolium) assays were performed.

Two different compounds were used as positive controls: 5% sodium dodecyl sulfate (SDS) (Invitrogen, Carlsbad, CA, USA) and 50 mM hydrogen peroxide (H_2_O_2_) (Sigma-Aldrich, St. Louis, MO, USA). SDS is a detergent commonly used to lyse cells and is thus expected to reduce cell viability. H_2_O_2_ causes damages to vital cellular components, such as mitochondria, and is commonly used as positive control of cell damage. Due to their different levels of toxicity when in contact with *S. cerevisiae* cells, each component had their own specific exposure time: 1 h exposure time for SDS and 30 min for H_2_O_2_.

To perform the MTT assay *S. cerevisiae* VL3 cultures were grown in YPD with different pure MC-LR concentrations: without toxin (control), 1 nM, 10 nM, 100 nM and 1 µM, during 4 h in 6 wells microplates (Nest Biotechnology, Jiangsu, China) (for easier manipulation).

After the exposure, 1 mL of the suspension from each well was harvested to 2 mL tubes and centrifuged for 5 min at 8000× *g* (Eppendorf 5415C Centrifuge). The supernatant was discarded and the cells were resuspended in 100 µL of PBS (phosphate-buffered saline) buffer. Then, 100 µL of freshly prepared MTT solution was added, and the tubes incubated in the dark in the climatic chamber with agitation for 2 h. Afterwards, the suspension was centrifuged for 5 min at 8000× *g* and the supernatant was discarded. The pellet was resuspended in 300 µL dimethyl sulfoxide (DMSO) (Thermo Fisher, Carlsbad, CA, USA) and 100 µL of each was inoculated in a 96 well plate (Sarstedt, Nümbrecht, Germany), performing 3 replicates in total for each treatment. The absorbance was read by a microplate spectrophotometer (Thermo Fisher LabSystems) at 570 nm with a reference wavelength of 690 nm.

The relative cell viability (%) was calculated using the following formula:

(1) $$\mathrm{relative}\;\mathrm{cell}\;\mathrm{viability}\;\left(\%\right)\;=\;\frac{\mathrm{average}\;\mathrm{absorbance}\;\mathrm{of}\;\mathrm{experimental}\;\mathrm{groups}\;}{\mathrm{average}\;\mathrm{absorbance}\;\mathrm{of}\;\mathrm{the}\;\mathrm{control}\;\mathrm{group}}\times \;100$$

### 5.3. Proteomic Analysis

#### 5.3.1. Protein Extraction and Sample Preparation

Yeast cells pellets (replicate samples, *n* = 3) were homogenized in Tris (100 mM), SDS 2% (*w*/*v*), dithiothreitol (0.1 M), pH 7.6 and protease inhibitors (complete protease cocktail tablets, Roche, Basel, Switzerland) with sonication (6 cycles of 5 s at 60 Hz). After 2 h incubation at room temperature, samples were heated (95 °C, 3 min) and clarified at 16,000× *g* for 20 min. The supernatants were recovered and total protein estimated at 280 nm. Proteins were digested following the filter aided sample preparation method described by [39], with centrifugal filter units with molecular weight cut-off of 30 kDa (MRCF0R030, Millipore, Billerica, MA, USA). Protein samples (40 µg protein) were alkylated with iodoacetamide and digested with trypsin (Roche, 03708985001) for 16 h at 37 °C, at an enzyme to protein ratio of 1:100 (*w*/*w*). Protein digests were recovered by centrifugal filtration, acidified with formic acid (FA) (10%, *v*/*v*), desalted, and concentrated by reversed phase extraction (C18 Tips 100 µL, Thermo Scientific, 87784). Before LC–MS/MS, the peptides were resuspended in 0.1% (*v*/*v*) FA to the concentration of 0.04–0.06 µg/µL as described by [40].

#### 5.3.2. LC-MS/MS and Protein Identification

The LC-MS/MS was carried out as described previously in a nano-LC coupled to a hybrid Ion trap mass spectrometer (Linear Trap Quadropole - LTQ Orbitrap Velos Pro–ETD, Thermo Scientific, Waltham, MA, USA) [40]. Peptides were separated by reverse phase chromatography on a 20 mm × 100 µm C18 precolumn followed by a 100 mm × 75 µm C18 column (NanoSeparations, Nieuwkoop, The Netherlands) in a linear gradient of acetonitrile (2% to 95% *v*/*v*) in FA (0.1% *v*/*v*), at a flow rate of 0.3 µL/min. Full scans were performed at 30,000 resolution at a range of 380–2000 *m*/*z*. The top 20 most intense ions were isolated and fragmented with collision induced fragmentation (CID) applying normalized collision energy of 30%, isolation width of 2.0, and activation time of 10 ms and a Q-value of 0.25. In total, 15 independent LC-MS/MS runs were performed from 15 biological samples (including replicates).

Proteins were identified by searching LTQ raw data against *Saccharomyces* sequences in UNIPROT database using SEQUEST algorithm (Proteome Discoverer software, version 1.4, Thermo Scientific, Waltham, MA, USA) and the X! Tandem algorithm in Scaffold (version Scaffold 4.3.4, Proteome Software, Portland, OR, USA). Peptides were accepted at a probability greater than 95.0% and proteins greater than 99.9%. MS and MS/MS tolerances were set to 10 ppm and 0.6 Da. Static and dynamic modifications were carbamidomethylation and oxidation, respectively. Trypsin was selected for protein cleavage allowing for one missed cleavage.

Functional analysis of proteins was performed with the bioinformatics tool STRING [27]. This analysis was performed by selecting the organism *Saccharomyces cerevisiae*; the sources of evidence text mining, experiments, databases, co-expression, neighborhood, gene fusion, co-occurrence; the interaction score medium confidence (0.400) and max number of interactors (1st shell) no more than 5.

### 5.4. Real-Time RT-qPCR Assays

#### 5.4.1. *Saccharomyces cerevisiae* Studied Genes and Primers Design

In this study, Real-Time RT-qPCR was used to provide quantitative measurements of gene expression. It was intended to determine how the expression of a particular gene changes in response to MC-LR exposure. Knowing that MC-LR causes oxidative DNA damages that are probably mainly repaired by the *S. cerevisiae* DNA BER system, the following genes were selected: *Apn1*, *Apn2*, *Rad27*, *Ntg1,* and *Ntg2* to evaluate their expression levels in cells exposed to different concentrations of MC-LR. The primers for *Ntg1*, *Ntg2* genes had been previously designed by [17], and primers for genes *Apn1*, *Apn2,* and *Rad27* were designed in this study (Table 3).

We selected the reference genes *Alg9* to be used as internal control [42] for posterior expression normalization.

#### 5.4.2. *Saccharomyces cerevisiae* RNA Extraction and Purification

To evaluate the alterations of the expression levels of the *S. cerevisiae* previously selected genes by Real-Time RT-qPCR, *S. cerevisiae* RNA was extracted using TRIzol method [17]. Afterwards RNA samples were purified using the High Pure RNA Isolation Kit (Roche), according to the manufacturer instructions. To confirm that purified RNA was free from DNA, the samples were subjected to a conventional PCR prior to RT-qPCR analyses.

#### 5.4.3. Evaluation of RT-qPCR Parameters

Before starting the Real-Time RT-qPCR assays to determine the expression levels, the optimal conditions for RT-qPCR performance had to be verified. The linear regression coefficient (R^2^) must be above 0.95% and efficiency must be between 80–115% [43]. The optimization method was based on the construction of standard calibration curves for Real-Time RT-qPCR using several dilutions of the DNA.

The RT-qPCR conditions were optimized for all the target genes: *Apn1*, *Apn2*, *Rad27*, *Ntg1*, *Ntg2,* and the reference gene *Alg9*, by confirming that reactions had a linear regression coefficient (R^2^) above 0.95 and the efficiency value was between 88% and 100%, as summarized in Table 4.

### 5.5. Statistics

Proteomic data was analyzed by ANOVA and Student’s t-test, using the program Multiple Experiment Viewer [44] for processing OMICs data. Significant differences were assumed for *p* < 0.05.

A normalization using the mathematical method developed by Pfaffl [45] was conducted to determine the relative expression levels of BER genes (*Apn1*, *Apn2*, *Rad27*, *Ntg1,* and *Ntg2*). Five independent assays were made to obtain more consistent results. The expression results where normalized using the ALG9 gene.

## Figures and Tables

**Table 1:** Relative cell viability (%) from the control of *S. cerevisiae* VL3 strain exposed to two positive controls (H_2_O_2_ or SDS) and to different MC-LR concentrations. The values are the average of three different biological assays (*n* = 3). Asterisks (*) represent significant differences from the control (*p* < 0.05; *t*-Test).

Condition	Cell Viability (%)
Control (cells grown in YPD)	100.0
With 50 mM H_2_O_2_	40.6 *
With 5% SDS	10.8 *
With 0.2 nM MC-LR	100.2
With 0.4 nM MC-LR	96.2
With 1 nM MC-LR	108.3
With 10 nM MC-LR	101.5
With 100 nM MC-LR	109.1
With 1 µM MC-LR	105.2

**Table 2:** Protein markers of yeast response to MC-LR. Expression values for the MC-LR concentrations tested and mechanisms associated. Asterisks (*) represents significant differences in respect to control for *p* < 0.05.

			MC-LR			
Mechanisms Associated ^(a)^	Proteins ^(b)^	Control	1 nM	10 nM	100 nM	1 µM
Protein abundance increases in response to DNA replication stress	ENO1	0.0019 ± 0.00024	0.0054 ± 0.00078 *	0.0038 ± 0.00233	0.0073 ± 0.00347	0.0031 ± 0.00005 *
	PIL1	0.0 ^(c)^	0.0005 ± 0.00005 *	0.0002 ± 0.00032	0.0006 ± 0.00033	0.0004 ± 0.0003 *
	TDH2	0.0009 ± 0.00103	0.0042 ± 0.00098 *	0.0017 ± 0.00291	0.0045 ± 0.00053 *	0.0036 ± 0.00038 *
	ARC1	0.0016 ± 0.00040	0.0014 ± 0.00013	0.0018 ± 0.00033	0.0011 ± 0.00032	0.0005 ± 0.00050 *
	TRR1 ≈ TRR2	0.0005 ± 0.00081	0.0031 ± 0.00122 *	0.0012 ± 0.00202	0.0027 ± 0.00075 *	0.0040 ± 0.00041 *
Nuclear Excision Repair Factor 2 (NEF2)	RAD23	0.0007 ± 0.00019	0.0006 ± 0.00029	0.0006 ± 0.00019	0.0012 ± 0.00010 *	0.0013 ± 0.00016 *
Gene upregulated in cancer	NOP2	0.0025 ± 0.00041	0.0010 ± 0.00023 *	0.0011 ± 0.00050 *	0.0012 ± 0.00044 *	0.0011 ± 0.00014 *
Responds to oxidative stress	MGE1	0.0012 ± 0.00034	0.0 *	0.0 *	0.0007 ± 0.00006	0.0009 ± 0.00009
Involved in actin cytoskeleton maintenance	TCP1	0.0	0.0003 ± 0.00029 *	0.0005 ± 0.00014	0.0002 ± 0.00016	0.0
Several ribosomal proteins	RPL36B	0.0034 ± 0.0011	0.0053 ± 0.00137	0.0109 ± 0.00463	0.00474 ± 0.00146	0.0073 ± 0.00186 *
	RPL32	0.0116 ± 0.00202	0.0065 ± 0.00136 *	0.0080 ± 0.00228	0.0077 ± 0.00093	0.0082 ± 0.00090
	RPL24A	0.0022 ± 0.00063	0.0009 ± 0.00081	0.0 *	0.0009 ± 0.00077	0.0005 ± 0.00086
	RPS6B	0.0099 ± 0.00124	0.0057 ± 0.00101 *	0.0099 ± 0.00190	0.0059 ± 0.00086 *	0.0069 ± 0.00189
	RPS20	0.0156 ± 0.00120	0.0100 ± 0.00488	0.0153 ± 0.00272	0.0080 ± 0.00186 *	0.0121 ± 0.00077 *
	RPS2	0.0011 ± 0.00031	0.0039 ± 0.00106 *	0.0021 ± 0.00079	0.0041 ± 0.00095 *	0.0029 ± 0.00068 *
	RPS23A	0.0	0.0020 ± 0.00116	0.0010 ± 0.00095	0.0025 ± 0.00064 *	0.0018 ± 0.00071 *
Activity in nuclear ribosomal-subunit export impaired by mild oxidative stress	RLI1	0.0005 ± 0.00013	0.0 *	0.0004 ± 0.00013	0.0 *	0.0001 ± 0.00018
Involved in ER to Golgi transport	SEC21	0.0	0.0001 ± 0.00020	0.0001 ± 0.00011	0.0003 ± 0.00003 *	0.0
	SAR1	0.0	0.0009 ± 0.00008 *	0.0	0.00053 ± 0.00046	0.0003 ± 0.00057
Decreases upon DNA replication stress	PNC1	0.0	0.0008 ± 0.00007 *	0.0003 ± 0.00050	0.0012 ± 0.00035 *	0.0009 ± 0.00010 *
	VPH1	0.0	0.0002 ± 0.00002 *	0.0001 ± 0.00013	0.0004 ± 0.00020	0.0001 ± 0.00016
Specific translational activator for the mitochondrial COX1 mRNA	MAM33	0.0016 ± 0.00019	0.0011 ± 0.00035	0.0012 ± 0.00020	0.0004 ± 0.00033 *	0.0013 ± 0.00050
Enzyme of the ‘de novo’ purine nucleotide biosynthetic pathway	ADE5,7	0.0	0.0001 ± 0.00014	0.0003 ± 0.00010 *	0.0001 ± 0.00011	0.0
Polypeptide release factor (eRF1) in translation termination	SUP45	0.0	0.0005 ± 0.00018 *	0.0001 ± 0.00025	0.0007 ± 0.00006 *	0.0002 ± 0.00030
Interaction with Top1p and nucleolar localization are negatively regulated by polyphosphorylation	NSR1	0.0078 ± 0.00074	0.0065 ± 0.00097	0.0091 ± 0.00155	0.0061 ± 0.00025 *	0.0061 ± 0.00052 *
Required for the assembly of actin and tubulins in vivo	CCT4	0.0001 ± 0.00024	0.0012 ± 0.00018 *	0.0002 ± 0.00041	0.0010 ± 0.00060	0.0006 ± 0.00025
Heat Shock Protein	HSP10	0.0072 ± 0.00106	0.0028 ± 0.00091 *	0.0059 ± 0.00213	0.0030 ± 0.00026 *	0.0044 ± 0.00085 *
Involved in glycolysis and gluconeogenesis	GPM1	0.0105 ± 0.00076	0.0081 ± 0.00131	0.0078 ± 0.00037 *	0.0079 ± 0.00037 *	0.0091 ± 0.00103
Involved in TCA cycle	FUM1	0.0	0.0001 ± 0.00022	0.0001 ± 0.00022	0.0003 ± 0.00003 *	0.0006 ± 0.00031
Catalyzes the oxidation of isocitrate to alpha-ketoglutarate	IDP1	0.0	0.0010 ± 0.00014 *	0.0	0.0010 ± 0.00028 *	0.0006 ± 0.00051

**Legend:**^(a)^ according to https://www.yeastgenome.org/; ^(b)^ protein full names are presented in Supplementary Table S2; ^(c)^ zero values in the table indicate that the protein was not detected in the experimental group.

**Table 3:** Description of primers for Base Excision Repair (BER) system genes and their functions.

Gene	Primer Sequence	Molecular Function	Primers Designed in
*Apn1*	F: 5’-TGG GTT TCT CCG CAG TAT-3’	AP endonuclease/3′-diesterases, enzymes whose function is to excise mutagenic and cytotoxic AP or 3′ phosphate/phosphoglycolate groups from DNA [36]	This study
	R: 5’-GCC TAT CCC TAA TTG CTC AC-3’		
*Apn2*	F: 5’-TGC TAA TGG GCG ACG TAA AT-3’		This study
	R: 5’-GGC GTG TCC GGA TTG ATA ATA-3’		
*Rad27*	F: 5’-CCG CAG CAA GTG AAG ATA TG-3’	5′→3′ exonuclease and a flap endonuclease [41]	This study
	R: 5’-CCA ACA CCT CTG ATG CTT TC-3’		
*Ntg1*	F: 5’-CAT TCC TGT AAC GGT TGC CT-3’	DNA glycosylases/AP lyases. These excise primarily oxidized pyrimidines [36]	[17]
	R: 5’-TTG TGT GGA ACC CAA CTG AA-3’		
*Ntg2*	F: 5’-AAC ACT GCA AAA AGG TTG GG-3’		[17]
	R: 5’-GAC CAA ATC CAA CCA AAA CG-3’		

**Table 4:** Threshold RT-qPCR reaction parameters of target and reference genes.

Gene		Annealing Temperature (°C)	Efficiency (%)	Slope	Y Intercept	R^2^
**BER genes**	*Apn1*	52	98	−3.361	24.083	0.997
	*Apn2*	52	100	−3.314	26.196	0.992
	*Rad27*	52	96	−3.416	24.524	0.998
	*Ntg1*	48	88	−3.649	25.863	0.993
	*Ntg2*	48	94	−3.484	21.605	0.993
**Reference gene**	*Alg9*	58	97	−3.398	23.906	0.989

## Supplementary Materials

The following are available online at https://www.mdpi.com/2072-6651/12/10/667/s1. Supplementary Table S1: List of all of *Saccharomyces cerevisiae* proteins identified by LC-MS/MS and respective relative expression values in all experimental groups. Supplementary Table S2: Expression values and ANOVA results of *Saccharomyces cerevisiae* differentially expressed proteins (DEPs).
